# Racial, Ethnic, and Education Differences in Age of Smoking Initiation Among Young Adults in the United States, 2002 to 2019

**DOI:** 10.1001/jamanetworkopen.2023.5742

**Published:** 2023-03-30

**Authors:** Alyssa F. Harlow, Rob McConnell, Adam M. Leventhal, Renee D. Goodwin, Jessica L. Barrington-Trimis

**Affiliations:** 1 Department of Population and Public Health Sciences, University of Southern California, Los Angeles; 2 Institute for Addiction Science, University of Southern California, Los Angeles; 3 Department of Epidemiology and Biostatistics, Graduate School of Public Health and Health Policy, The City University of New York, New York

## Abstract

This cross-sectional study examines differences by race and ethnicity and education in smoking prevalence and age of smoking initiation among young adults in the US between 2002 and 2019.

## Introduction

Among US young adults who smoke cigarettes, the proportion who initiated smoking during young adulthood (vs adolescence) doubled from 20.6% in 2002 to 42.6% in 2018.^1^ Later age of smoking initiation is associated with lower risk of smoking-related harms,^2^ and this shift toward young adult smoking initiation is likely attributed to tobacco control efforts aimed at reducing youth smoking.^3^ However, it is unclear whether trends toward later smoking initiation have occurred equitably across demographic groups. We examined differences by race and ethnicity and education in smoking prevalence and age of smoking initiation among US young adults between 2002 and 2019.

## Methods

This cross-sectional study was exempt from federal regulations for the protection of human research participants (45 CFR §46) and followed the STROBE reporting guideline. Data were from the National Survey on Drug Use and Health (NSDUH), an annual cross-sectional survey representative of the US civilian noninstitutionalized population.^4^ Analyses were restricted to adults aged 21 to 25 years.

Participants reported their age, gender, race and ethnicity, education, ever cigarette use (yes or no), ever daily smoking (yes or no), age at which they first smoked, and age at which they first smoked daily. We created dichotomous variables for age of initiation (during young adulthood [aged 18 to 25 years] vs adolescence [aged less than 18 years]).

Analyses were weighted using NSDUH survey weights. Across survey years (2002 to 2019), we calculated prevalence of ever and daily smoking, smoking initiation during young adulthood (vs adolescence; among ever smokers), and daily smoking initiation during young adulthood (vs adolescence; among ever daily smokers), stratified by education and race and ethnicity. To examine time trends, we fit logistic regression models with continuous year as the independent variable, adjusting for gender and age, and scaling odds ratios to represent change per 5 years. We examined time × education and time × race and ethnicity interactions (eMethods in Supplement 1). Analyses were conducted using SAS 9.4 (SAS Institute) with statistical significance defined as α = .05 (2-tailed).

## Results

The sample included 187 821 young adults (50.7% female; 19.4% Hispanic, 13.7% non-Hispanic Black; 58.7% non-Hispanic White). There were 60.0% with at least some college education, 28.1% with high school education, and 11.9% with less than high school education.

**Figure 1.**
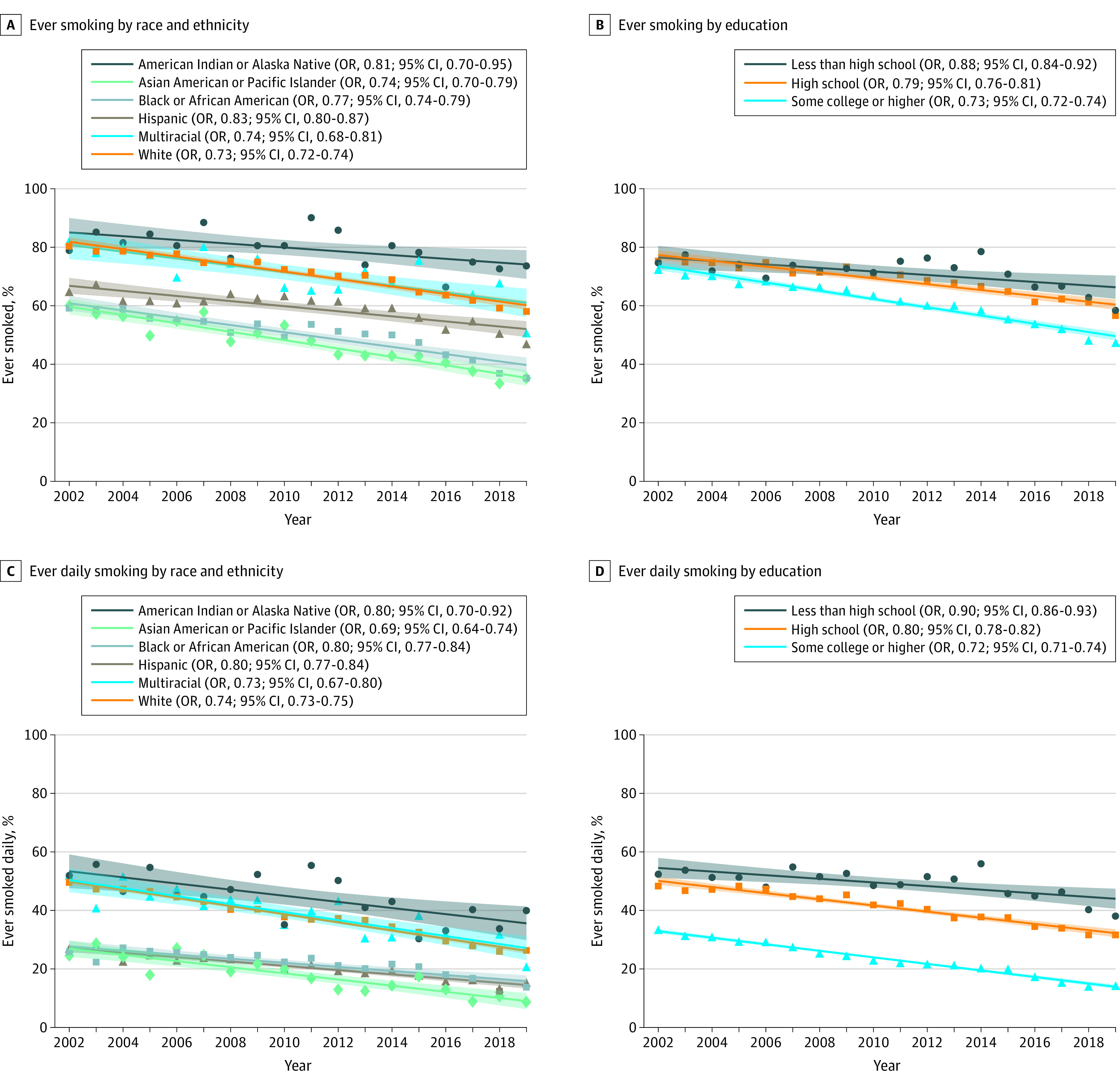
Trends in Ever and Daily Smoking by Race and Ethnicity and Education Among Young Adults Aged 21 to 25 Years in the United States, 2002 to 2019 Ever smoking prevalence stratified by race and ethnicity (A) and education (B); ever daily smoking prevalence stratified by race and ethnicity (C) and education (D). Odds ratios (OR) and 95% CIs from logistic regression models with year as continuous independent variable adjusted for gender and age, scaled to 5-year units. Bands indicate 95% CI from linear trend line.

The prevalence of ever (Figure 1A and 1B) and daily (Figure 1C and 1D) smoking declined significantly from 2002 to 2019 for all education and racial and ethnic groups, and was lowest among those with at least some college education, and for Asian and Black young adults. Per-5-year decreases in the prevalence of ever and daily smoking were significantly slower for participants with less than high school or high school education (eg, trend in ever smoking among less than high school: OR, 0.88 [95% CI, 0.84-0.92] vs at least some college education: OR, 0.73 [95% CI, 0.72-0.74]) and Black and Hispanic (vs White) young adults (interaction *P* values < .001).

**Figure 2.**
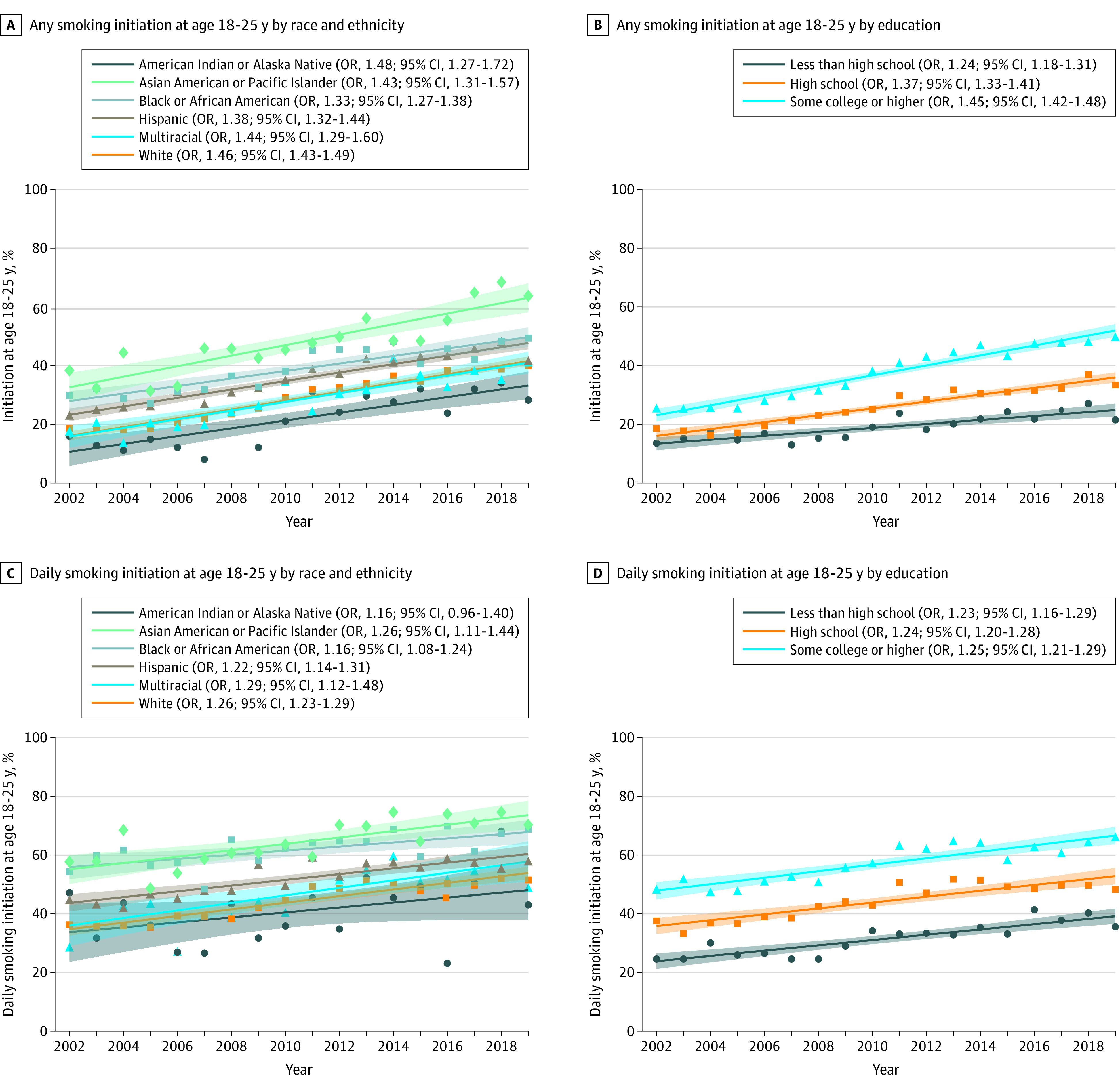
Trends in Smoking Initiation During Young Adulthood by Race and Ethnicity and Education Among Young Adults Aged 21 to 25 Years in the United States, 2002-2019 Proportion of ever cigarette smokers who began smoking at ages 18 to 25 years stratified by race and ethnicity (A) and education (B); proportion of ever daily cigarette smokers who began daily smoking at ages 18 to 25 years stratified by race and ethnicity (C) and education (D). Odds ratios (OR) and 95% CIs from stratified logistic regression models with year as continuous independent variable adjusted for gender and age, scaled to 5-year units. Bands indicate 95% CI from linear trend line.

Among 124 507 ever smokers and 63 757 ever daily smokers, the proportion who initiated ever (Figure 2A and 2B) and daily (Figure 2C and 2D) smoking during young adulthood (vs adolescence) increased significantly from 2002 to 2019 among all education groups and nearly all racial and ethnic groups, and was highest among those with at least some college education and Asian and Black young adults. Per-5-year increases in the prevalence of ever smoking initiation during young adulthood were significantly slower for participants with less than high school or high school education (eg, trend in any smoking initiation at age 18-25 years among those with less than high school: OR, 1.24 [95% CI, 1.18-1.31] vs some college: OR, 1.45 [95% CI, 1.42-1.48]) and Black (vs White) participants (interaction *P* values < .001). There were no differences in age of daily smoking initiation trends by education or race and ethnicity.

## Discussion

Declines in smoking prevalence and increases in the age of smoking initiation occurred more slowly for young adults with less formal education, widening existing education disparities between 2002 and 2019. Black young adults had lower smoking prevalence and older age of smoking initiation than White young adults. However, declines in smoking prevalence and increases in the age of smoking initiation occurred more slowly for this group. This study is limited by self-reported recalled age of smoking initiation. Targeted marketing, availability of menthol cigarettes, economic inequalities, and racism and discrimination may have contributed to slower decreases in smoking prevalence and slower increases in the age of smoking initiation for minoritized racial and ethnic young adults as well as those with less education.^5^ It is also possible that public health efforts aimed at reducing youth smoking, including prohibition of youth-targeted tobacco marketing, Tobacco-21 laws, and education campaigns, may not be benefiting all populations equally.^1,3^

## Notes

Supplement 1.
eMethods.

eReference

Supplement 2.
Data Sharing Statement
