# Supplementary material for: Racial, Ethnic, and Education Differences in Age of Smoking Initiation Among Young Adults in the United States, 2002 to 2019
Source: JAMA Netw Open. 2023 Mar 30;6(3):e235742. doi: 10.1001/jamanetworkopen.2023.5742 (PMC10064249; doi:10.1001/jamanetworkopen.2023.5742)
Supplement: Supplement 1. — eMethods. eReference [file jamanetwopen-e235742-s001.pdf]

Supplemental Online Content

Harlow AF, McConnell R, Leventhal AM, Goodwin RD, Barrington-Trimis JL. Racial, ethnic, and education differences in age of smoking initiation among young adults in the United States, 2002 to 2019. *JAMA Netw Open*. 2023;6(3):e235742. doi:10.1001/jamanetworkopen.2023.5742

eMethods.

eReferences

This supplemental material has been provided by the authors to give readers additional information about their work.

© 2023 Harlow AF et al. *JAMA Network Open.*

eMethods

Data Source and Sample

This study used 18 years of survey data from the NSDUH public data portal. NSDUH is a nationally representative annual cross-sectional survey of civilian non-institutionalized individuals ≥12 years old in the US. Details on the data collection methods and response rates of NSDUH have been reported previously.[1] We analyzed data from 2002-2019 survey years. Due to changes in survey methodology after 2019 (i.e., due to COVID-19) we did not include 2020 data. The analysis was restricted to 187,821 young adults 21-25 years old. The per-year sample size ranged from 8,223 in 2014 to 11,774 in 2011.

Measures

Ever Cigarette Smoking and Age of Smoking Initiation

Participants were asked whether they had ever smoked all or part of a cigarette. Those who responded yes were classified has having ever smoked cigarettes, and those who responded no were classified as having never smoked cigarettes. Participants who reported ever smoking were asked to report the age at which they first smoked all or part of a cigarette. We created a dichotomous variable for smoking initiation during young adulthood (18-25y) versus adolescence (<18y).

Daily Cigarette Smoking and Age of Daily Smoking Initiation

Participants who had ever smoked a cigarette were asked if there was ever a period in their life when they smoked cigarettes every day for at least 30 days. Those who responded yes were classified as having ever smoked cigarettes daily, and those who responded no were classified as having never smoked cigarettes daily. Participants who reported ever smoking cigarettes daily were asked to report the age at which they first started

© 2023 Harlow AF et al. *JAMA Network Open.*

smoking cigarettes daily. We created a dichotomous variable for first transitioning to daily smoking during young adulthood (18-25y) versus adolescence (<18y).

Sociodemographic Characteristics

Participants reported their age (21, 22-23, 24-25 years), gender (male female), race/ethnicity (non-Hispanic [NH] White, NH Black or African American, NH Native American or Alaska Native, NH Asian, Native Hawaiian, or Pacific Islander [AAPI; NH Native Hawaiian or Pacific Islander and NH Asian were assessed separately and collapsed in analysis], NH Multiracial, Hispanic), highest level of education (<high school, high school graduate or general educational diploma, some college or higher [some college and college degree or higher were assessed separately and collapsed in analysis]).

Statistical Analysis

All analyses are weighted using NSDUH survey sample weights to produce nationally representative estimates. We calculated the prevalence of ever smoking and ever daily smoking among the full sample of young adults for each survey year (2002-2019), stratified by race/ethnicity and education. We then calculated the proportion of young adult ever smokers who first initiated smoking during young adulthood (versus adolescence), and the proportion of young adult ever daily smokers who first transitioned to daily smoking during young adulthood (versus adolescence) for each survey year (2002-2019), stratified by race/ethnicity and education. To examine linear time trends for each smoking indicator, we fit logistic regression models with continuous year as the predictor stratified by race/ethnicity and education. Models adjusted for age and gender, and were scaled so that odds ratios and 95% confidence intervals represented the change in odds of each smoking indicator for every five-year increase. To determine whether linear time trends differed by race/ethnicity or education, we included an interaction term between continuous time x each sociodemographic characteristic. For interactions that were significant at the alpha=0.05 level, we further tested group-specific two-way interactions, using White as the reference group for race/ethnicity analyses, and some college or higher as the reference group for education analyses.

© 2023 Harlow AF et al. *JAMA Network Open.*

eReference

1. U.S. Department of Health and Human Services, Substance Abuse and Mental Health Services Administration, Center for Behavioral Health Statistics and Quality. National Survey on Drug Use and Health 2002-2019. Published 2018. Accessed September 30, 2022. https://www.datafiles.samhsa.gov/

© 2023 Harlow AF et al. *JAMA Network Open.*
