# Supplementary material for: Racial, Ethnic, and Education Differences in Age of Smoking Initiation Among Young Adults in the United States, 2002 to 2019
Source: JAMA Netw Open. 2023 Mar 30;6(3):e235742. doi: 10.1001/jamanetworkopen.2023.5742 (PMC10064249; doi:10.1001/jamanetworkopen.2023.5742)
Supplement: Supplement 2. — Data Sharing Statement [file jamanetwopen-e235742-s002.pdf]

Data Sharing Statement

Harlow. Racial, Ethnic, and Education Differences in Age of Smoking Initiation Among Young Adults in the United States, 2002 to 2019. *JAMA Netw Open*. Published March 30, 2023. doi:10.1001/jamanetworkopen.2023.5742

Data
Data available: Yes
Data types: Deidentified participant data
How to access data: This manuscript uses publicly available NSDUH data, which is available from the following website: https://www.datafiles.samhsa.gov/dataset/national-survey-drug-use-and-health-2019-nsduh-2019-ds0001
When available: With publication

Supporting Documents
Document types: None

Additional Information
Who can access the data: NSDUH data are publicly available to any person.
Types of analyses: NSDUH data are publicly available for any purpose.
Mechanisms of data availability: NSDUH are available at the following website: https://www.datafiles.samhsa.gov/dataset/national-survey-drug-use-and-health-2019-nsduh-2019-ds0001
